# Electrochemical Machining of Micro-Pit Arrays on a GH4169 Alloy with a Roll-Print Mask Using a C_6_H_5_Na_3_O_7_-Containing NaNO_3_ Mixed Electrolyte

**DOI:** 10.3390/mi15101253

**Published:** 2024-10-12

**Authors:** Ge Qin, Shiwei Li, Meng Li, Haoyu Peng, Shen Niu, Xinchao Li, Huan Liu, Liang Yan, Pingmei Ming

**Affiliations:** School of Mechanical and Power Engineering, Henan Polytechnic University, Jiaozuo 454000, China; lswmzsx@163.com (S.L.); 18339165839@163.com (M.L.); mkgpeng1520@163.com (H.P.); ns2019@hpu.edu.cn (S.N.); hpulixinchao@163.com (X.L.); liuhuan@hpu.edu.cn (H.L.); yanliang@hpu.edu.cn (L.Y.); mingpingmei@163.com (P.M.)

**Keywords:** roll-print mask electrochemical machining, GH4169 alloy, C_6_H_5_Na_3_O_7_-containing NaNO_3_ mixed electrolyte, micro-pit arrays, additive

## Abstract

GH4169 alloy, a nickel-based superalloy known for its excellent high temperature resistance, corrosion resistance, mechanical properties, and high-temperature tribological properties, is widely used in industrial applications, such as in gas turbines for space shuttles and rocket engines. This study addresses the issue of electrolyte product residue in the electrochemical machining process of a GH4169 alloy by utilizing a C_6_H_5_Na_3_O_7_-containing NaNO_3_ new mixed electrolyte. Comparative investigations of the electrochemical behavior and electrolyte product removal mechanisms at different concentrations of C_6_H_5_Na_3_O_7_ additive in NaNO_3_ solutions were conducted. The effects of additives, applied voltage, and the rotating speed of the cathode tool on the processing performance of micro-pit arrays on a GH4169 alloy were analyzed. The results indicate that the mixed solution containing C_6_H_5_Na_3_O_7_ significantly improves the localization and geometric morphology of the micro-pits compared to a single NaNO_3_ solution. The optimal electrochemical machining parameters were identified as 0.5 wt% C_6_H_5_Na_3_O_7_ + 10 wt% NaNO_3_ mixed electrolyte, 12 V applied voltage, and 0.1 r/min rotating speed of the cathode tool. Under these conditions, high-quality micro-pit arrays with an average diameter of 405.85 μm, an average depth of 87.5 μm, and an etch factor (EF) of 1.67 were successfully fabricated, exhibiting excellent morphology, localization, and consistency.

## 1. Introduction

Compared with traditional metal materials, the GH4169 alloy exhibits excellent thermal hardness, high-temperature fatigue resistance, and high-temperature mechanical and tribological properties, and this makes it widely used in nuclear energy, aerospace, and petrochemical industries [1,2]. In these applications, the surfaces of workpieces are often required to have micro-structure arrays with high precision and different shapes, sizes, and arrangements to meet various application requirements such as wear reduction and heat dissipation performance. The traditional machining [3,4] and the non-traditional machining methods, such as abrasive water jet machining [5,6], ultrasonic machining [7,8], electrical discharge machining (EDM) [9,10,11], and laser machining [12,13,14,15], have been explored to enhance surface machining precision. These methods have shown potential for improving the processing of surface micro-structure arrays and meeting machining precision requirements. However, they also cause various surface defects such as burrs, micro-cracks, and recast layers due to mechanical forces or instantaneous high-temperature effects. These defects can negatively impact the performance, reliability, and lifespan of GH4169 alloy workpieces. The electrochemical machining (ECM) technique can avoid the occurrence of these defects and offers a promising solution to this problem. It is a processing method that utilizes the principle of electrochemical reactions to achieve the micro-etching of materials and processing of micro-structure arrays on the surface of anode workpieces [16,17]. Several researchers have developed composite electrochemical machining methods such as jet electrochemical machining [18], through-mask electrochemical micromachining (TMEMM) [19] and electrochemical mill-grinding [20,21] by changing process parameters or combining traditional machining techniques. Wang et al. [22] successfully machined a sharp edge deep groove with a depth of 1.208mm on the surface of Inconel 718 using a 10 wt% NaNO_3_ single-electrolyte solution, based on the advantages of jet electrochemical machining combined with milling processes. Zhai et al. [23] successfully processed the micro-pit with a diameter of 167.77 um, a depth of 79.62 um, and an EF of 2.35 on the surface of the workpiece by using the through-mask electrochemical micromachining method and introducing the acoustic wave stirring process. Niu et al. [24] employed a machining method combining electrochemical grinding and the electrochemical milling process known as electrochemical mill-grinding, using Inconel 718 with good high-temperature mechanical properties as the anode workpiece and a 10 wt% NaNO_3_ single solution as the electrolyte. By designing a new machining tool, they managed to produce groove structures with an averagely smooth side wall flatness and reduced roughness during the finishing stage. These methods have shown high precision and good localization in the micro-structure array fabrication on the workpiece surface.

However, nickel-based superalloys are prone to forming insoluble electrolytic products during the ECM process due to their complex material composition. The insoluble electrolytic products are easily retained on the processed surface, making it challenging to remove them solely through high-speed electrolyte flow. To address this issue, researchers [25,26] have investigated the addition of various additives or complexing agents to a traditional single electrolyte. They explored the effects of these additives in the ECM of nickel-based superalloys, aiming to reduce the formation of electrolytic products and improve machining accuracy. Researchers indicate that the use of composite electrolyte solutions containing complexing agents can effectively reduce electrolytic products in ECM through complexation. Mariem et al. [27] investigated the anodic dissolution behavior of forged Inconel 718 in a mixed solution of sulfosalicylic acid and NaNO_3_, demonstrating a significant reduction in electrolytic products on the Inconel 718 surface. Wang et al. [28] employed pulse electrochemical machining, using a C_6_H_5_Na_3_O_7_ solution as the electrolyte and NaNO_3_ solution as a control. Their findings revealed that the C_6_H_5_Na_3_O_7_ electrolyte could delay the formation and hydrolysis of hydroxide products, resulting in an improved machined surface for Inconel 718. Sodium citrate (C_6_H_5_Na_3_O_7_) is an organic acid salt with strong complexing capabilities, commonly used as a complexing agent, buffer, emulsifier, and stabilizer [29,30]. Compared with traditional acidic or toxic organic solvent additives, C_6_H_5_Na_3_O_7_ is an environmentally friendly alternative, offering advantages such as low cost, environmental friendliness, and non-corrosiveness. These properties make it a promising additive with broad application potential. However, the existing literature about TMEMM is scarce when it comes to mitigating the challenges associated with the formation of insoluble electrolytic products. In this paper, C_6_H_5_Na_3_O_7_ additive was integrated into the electrolyte to enhance the machining precision and quality of the micro-pit arrays on the GH4169 alloy surface. Therefore, in order to effectively improve the retention of electrolytic products on the GH4169 alloy surface in the single solution, the environmentally friendly sodium citrate additive will be introduced into the sodium nitrate solution to reduce the production of electrolytic products and improve the machining quality and accuracy of the micro-structure arrays on the GH4169 alloy surface with its excellent complexing ability.

Roll-print mask electrochemical machining using a linear cathode is a machining method using a cathode tool fixed with a linear cathode and a flexible movable mask for electrochemical dissolution machining. It has a higher machining accuracy than the rotating through-mask electrochemical transferring technology with annular cathode proposed by Shen et al. [31] (EF = 0.78). Building on roll-print mask electrochemical machining using the linear cathode previously developed by our research group [32,33], C_6_H_5_Na_3_O_7_ is introduced as an additive into a NaNO_3_ electrolyte to create the mixed electrolyte for the TMEMM of micro-structure arrays on the GH4169 alloy surface. To further understand the dissolution characteristics of the GH4169 alloy in the mixed electrolyte and its impact on the surface morphology, a comparative analysis is conducted on the alloy’s dissolution behavior in electrolytes containing varying concentrations of C_6_H_5_Na_3_O_7_. Through these comparative experiments, the optimal concentration of the C_6_H_5_Na_3_O_7_ is identified, the reaction mechanism is explored, and the process parameters are optimized.

## 2. Experiment

### 2.1. Specimen Preparation

In this study, the workpiece materials are GH4169 alloy and 304 stainless steel (304 SS). Cube samples with dimensions of 30 mm × 20 mm × 1 mm were prepared. The electrochemical test and TMEMM experiment were carried out with GH4169 alloy as the anode workpiece, while the 304 SS was used for comparison experiments. Table 1 presents the main components of the anode workpieces. The primary elements in both GH4169 alloy and 304 SS are Ni, Cr, and Fe, along with small amounts of Mn, Si, C, and other elements. Additionally, GH4169 alloy contains special elements such as Nb, Mo, Ti, and Al, making its composition more complex than that of 304 stainless steel.

### 2.2. Experiments

**Figure 1:**
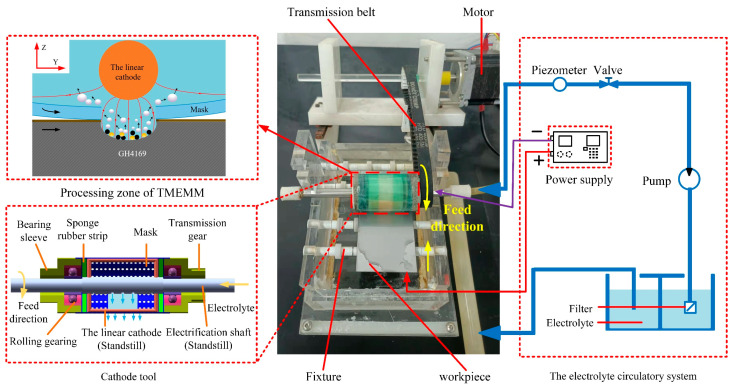
The schematic diagram of the roll-print mask electrochemical machining with the linear cathode experimental system.

TMEMM experiments were carried out using a self-developed experimental platform for roll-print mask electrochemical machining with the linear cathode (as shown in Figure 1). The main fabrication conditions and process parameters of micro-pit arrays are shown in Table 2. The platform consists of a processing unit, transmission unit, electrolyte circulation unit, control unit, and power supply unit. In this study, a GH4169 alloy specimen with dimensions of 30 mm × 20 mm × 1 mm was employed as the anode workpiece, and a linear metal wire with a diameter of 300 μm was utilized as the machining cathode, mounted on the cathode tool. During the rotating and machining of the cathode tool, the position of the linear cathode remained fixed. A polyimide (PI) sheet with a thickness of 100 μm was chosen as a flexible active mask, and micro-porous array patterns were fabricated on its surface using a femtosecond laser processing method. The inter-electrode gap was set to 100 μm, matching the thickness of the mask. A NaNO_3_ solution mixed with C_6_H_5_Na_3_O_7_ was selected as the electrolyte for machining experiments on the GH4169 alloy surface. On this basis, at least 18 sets of the exploration experiments of process parameters, such as the concentration of the additives, the applied voltage, and the rotating speeds of cathode tool, were carried out. Under the same experimental conditions, three sets of repetitive verification experiments were completed to determine the optimal process parameters for processing the micro-structure arrays on the GH4169 alloy surface.

### 2.3. Electrochemical Tests

The polarization curves in NaNO_3_ mixed with different concentrations of C_6_H_5_Na_3_O_7_ for GH4169 alloy were determined using a three-electrode configuration system on an electrochemical workstation (CHI604E, CH Instruments, China). A platinum electrode served as the auxiliary electrode (CE) and a saturated calomel electrode was used as the reference electrode (RE). The working electrode (WE) was a GH4169 alloy specimen with a size of 5 mm × 5 mm × 5 mm. All tests were conducted at a 10 mV/s scanning rate and with a −1.5 to 3 V voltage range.

After the machining experiments, the micro-pit arrays morphology on the GH4169 alloy surface was examined using scanning electron microscopy (SEM, Carl Zeiss NTS GmbH, Germany).

## 3. Results and Discussion

### 3.1. Electrochemical Characterization in the C_6_H_5_Na_3_O_7_-Containing NaNO_3_ Mixed Electrolyte of GH4169 Alloy

Appropriate electrolyte composition parameters are crucial for studying the anodic dissolution characteristics of the GH4169 alloy in different electrolytes. The influence of different electrolyte components on the electrochemical dissolution process of the GH4169 alloy were investigated by measuring polarization curves using the electrochemical workstation. This approach aimed to assess the feasibility of using NaNO_3_ as the primary electrolyte and to determine the optimal concentration of C_6_H_5_Na_3_O_7_ as the additive.

**Figure 2:**
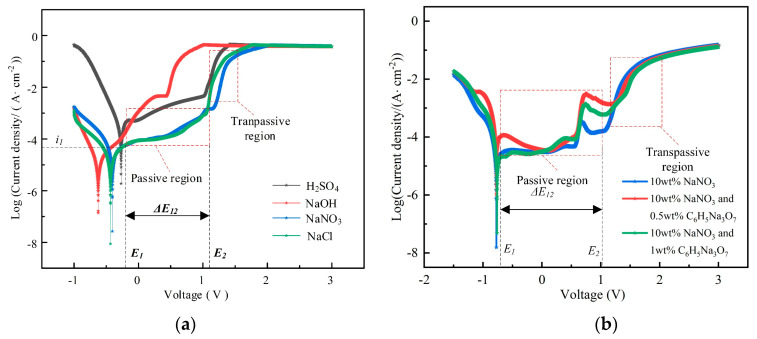
The measurement of polarization curves. (**a**) In the various electrolytes and (**b**) in the C_6_H_5_Na_3_O_7_-containing NaNO_3_ mixed electrolyte.

Figure 2 illustrates the influence of various electrolyte compositions on the electrochemical dissolution characteristics of the GH4169 alloy. There are obvious passivation regions in the polarization curves of the GH4169 alloy in 10 wt% H_2_SO_4_, NaCl, and NaNO_3_ solutions. In contrast, due to the strong corrosion of NaOH solution, discernible passivation regions are not observed in the corresponding polarization curves (as shown in Figure 2a). A comparative analysis of the polarization curves in 10 wt% H_2_SO_4_, NaOH, NaCl, and NaNO_3_ solutions reveals that the curve in 10 wt% NaNO_3_ solution displays the longer passivation interval, with smooth and stable over-passivation abilities. This is because both SO_4_^2−^ and NO_3_^−^ exhibit passivation abilities, but NO_3_^−^ demonstrates a stronger passivation effect, leading to a more stable passivation film [33]. Consequently, the passivation film formed in the non-machined zone in the NaNO_3_ solution is more stable, providing enhanced protection and reduced stray corrosion. Therefore, the 10wt% NaNO_3_ solution is selected as the primary electrolyte for TMEMM.

Figure 2b shows the impact of NaNO_3_ solutions with varying concentrations of C_6_H_5_Na_3_O_7_ on the electrochemical dissolution process of the GH4169 alloy. The polarization curves in NaNO_3_ solutions containing different concentrations of C_6_H_5_Na_3_O_7_ exhibit distinct passivation and over-passivation regions. The passivation region is extended in a NaNO_3_ solution containing 0.5 wt% C_6_H_5_Na_3_O_7_, indicating that the GH4169 alloy forms a more stable passivation film in this solution. This improved passivation helps protect the non-machined zone and reduces stray corrosion. Consequently, for subsequent experiments, the NaNO_3_ solution containing 0.5 wt% C_6_H_5_Na_3_O_7_ is chosen as the electrolyte for TMEMM to facilitate the production of high-quality and uniform micro-pit arrays.

### 3.2. The Optimal Process Parameters for the TMEMM of the GH4169 Alloy in the C_6_H_5_Na_3_O_7_-Containing NaNO_3_ Mixed Electrolyte

The appropriate process parameters are crucial for achieving the high-quality and precision machining of micro-pit arrays on the GH4169 alloy surface in a sodium nitrate solution with a sodium citrate additive. The selection of process parameters, including the concentration of the additive, the applied voltage, and the speed of the rotating cathode, is based on a systematic approach. Initially, we conducted a comprehensive review of the existing literature to determine the typical range of parameters used in similar studies. Subsequently, we carried out a series of preliminary experiments to assess the feasibility and effectiveness of different parameter ranges in our specific experimental setup.

#### 3.2.1. Influence of the Additive

**Figure 3:**
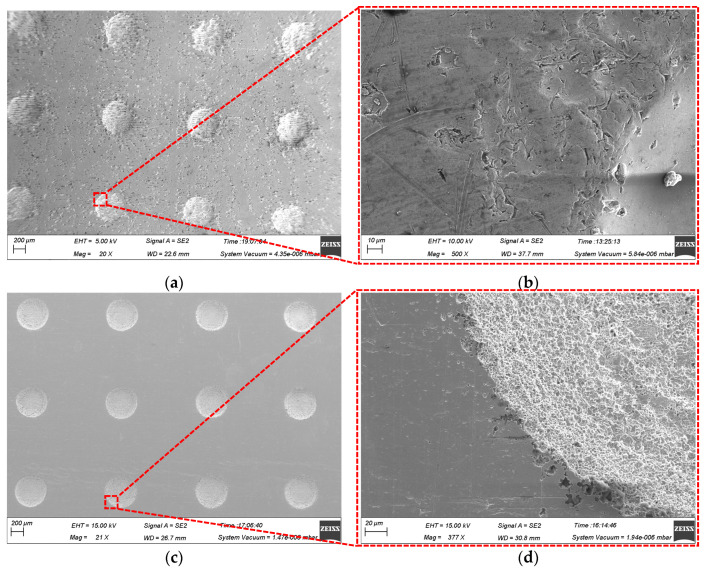
The surface morphology of the GH4169 alloy and 304 SS in 10 wt% NaNO_3_ electrolytes. (**a**) Macroscopic morphology of GH4169 alloy; (**b**) micro-structure arrays of GH4169 alloy; (**c**) macroscopic morphology of 304 SS; and (**d**) micro-structure arrays of 304 SS.

Figure 3 illustrates the surface morphology of the micro-pit arrays on the GH4169 alloy and 304 SS under the machining conditions of a 300 μm mask hole diameter, 10 wt% NaNO_3_ electrolyte, 12 V applied voltage, and 0.1 r/min rotating speed of the cathode tool. The micro-pit arrays on the GH4169 alloy surface exhibit uneven bottom and poor roundness at the entrances (as shown in Figure 3a,c), while those of the 304 SS are relatively flat with good roundness and minimal stray corrosion. This is attributed to the more complex element composition of the GH4169 alloy compared to 304 SS. During TMEMM processing, the accumulation of electrolytic products at the bottom of the micro-pit arrays in the GH4169 alloy impedes mass transfer within the flow and electric fields, severely affecting the formation of the micro-pit arrays.

Figure 3b,d reveal significant stray corrosion in the non-machined zone of the GH4169 alloy during TMEMM processing, resulting in extremely uneven surfaces. In contrast, 304 SS shows almost no stray corrosion in the non-machined zone. This difference is due to the features of the passivation film formed on the GH4169 alloy in NaNO_3_ solution at low current density, which is loose and porous [34]. This type of passivation film provides weak protection, allowing ions to penetrate and react with the underlying alloy. Consequently, the surface beneath the passivation film on the GH4169 alloy remains susceptible to partial corrosion. Conversely, 304 SS exhibits superior passivation in NaNO_3_ electrolyte, with a dense and uniform passivation film that offers excellent protection and effectively prevents stray corrosion at low current density.

**Figure 4:**
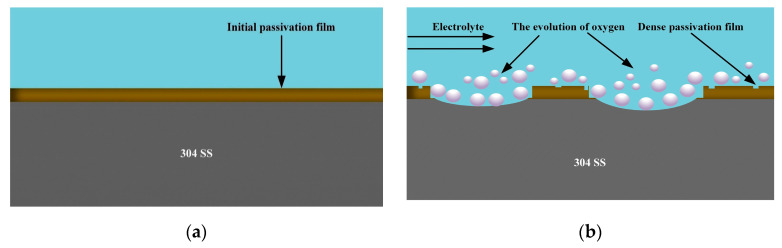
The dissolution characteristic model of 304 SS at low current density [34]. (**a**) Initial surface of 304 SS workpiece and (**b**) 304 SS surface under low current density.

**Figure 5:**
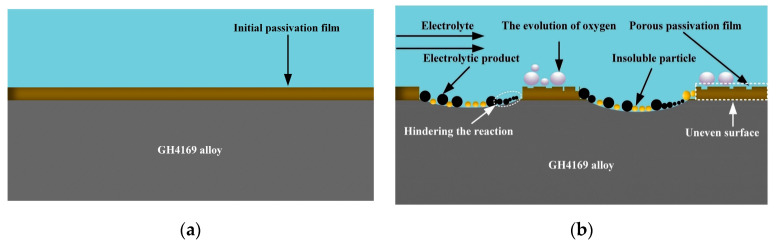
The dissolution characteristic model of the GH4169 alloy at low current density [34]. (**a**) Initial surface of GH4169 alloy workpiece and (**b**) GH4169 alloy surface under low current density.

Figure 4 and Figure 5 show the electrochemical dissolution process of the 304 SS and GH4169 alloy in 10 wt% NaNO_3_ electrolytes at low current density, respectively. Through comparative analysis, in the initial stage, there is an initial passivation film on the surface of 304 SS and the GH4169 alloy. With the progress of electrochemical reaction and the breakage of the initial passivation film, a new passivation film was reformed on the surface of the 304 SS and GH4169 alloy at low current density, accompanied by the generation of a multitude of bubbles. Compared with 304 SS, the new passivation film on the GH4169 alloy surface belongs to the loose porous passivation film, which has poor corrosion resistance and easily produces black insoluble electrolytic products and particles. At the same time, with the operation of the electrolyte circulation system, insoluble electrolytic products gradually accumulate in the inter-electrode gap, the solution will become more and more turbid, and the conductivity distribution will become uneven in the inter-electrode gap, which may lead to short circuiting or electrode burning. Therefore, with the purpose of effectively reducing the influence of insoluble electrolytic products on the electrochemical reaction process, the C_6_H_5_Na_3_O_7_ with complexing effects will be introduced in subsequent experiments to form the mixed electrolyte with NaNO_3_ solution, with the intention of realizing the exploration of the complexation mechanism between C_6_H_5_Na_3_O_7_ and insoluble electrolytic products and the high-precision machining of micro-pit arrays on the GH4169 alloy surface.

In the process of roll-print mask electrochemical machining, material removal occurs through an electrochemical reaction. The GH4169 alloy workpiece is used as the anode and dissolved into the electrolyte as M^n+^ metal cations (where M^n+^ mainly refers to Fe^2+^, Ni^2+^, Cr^3+^, etc.). The main metal cations at the anode and oxygen are produced as follows:

(1) $$M^{0}\;\to \;M^{n}++ne^{-}$$

(2) $$2H_{2}O\;\to \;4H^{+}+4e^{-}+O_{2}$$

The cathode primarily undergoes a hydrogen evolution reaction, which is as follows:

(3) $$2H_{2}O+2e^{-}\;\to \;H_{2}+2OH^{-}$$

(4) $$2H^{+}+2e^{-}\;\to H_{2}\;$$

As OH- migrates towards M^n+^ due to electrostatic attraction, intermediate products such as M(OH)_n_ are formed in the NaNO_3_ solution. As the electrochemical reaction progresses, M(OH)_n_ continues to decompose, producing water-insoluble oxides such as NiO, Cr_2_O_3_, Fe_2_O_3_, Nb_2_O_5_, and MoO_3_. The electrochemical reactions involved in the formation of these insoluble products are as follows [30]:

(5) $$M^{n+}+nOH^{-}=M(OH{)}_{n}$$

(6) $$mM(OH{)}_{n}=M_{m}O_{\frac{nm}{2}}+\frac{nm}{2}H_{2}O$$

When the NaNO_3_ solution is employed as the electrolyte for the electrochemical reactions, flocculent insoluble products are commonly observed. However, by utilizing the C_6_H_5_Na_3_O_7_-containing NaNO_3_ mixed electrolyte, the formation of these insoluble electrolytic products is significantly reduced. This can be attributed to the citrate ion (Cit^3−^) present in the C_6_H_5_Na_3_O_7_, which competes with the hydrophilic OH- group for metal cations, forming a water-soluble M(Cit^3−^)_a_ complex. These complexes are rapidly removed from the electrochemical processing zone by the electrolyte flow. Therefore, the use of C_6_H_5_Na_3_O_7_-containing NaNO_3_ mixed electrolyte enhances the removal of electrolytic products during the electrochemical reactions. The primary anodic reaction equation in this electrolyte system is expressed by the following equation [28]:

(7) $$M^{n+}+a(Cit^{3-})\;↔\;M(Cit^{3-}{)}_{a}$$

(8) $$M(OH{)}_{n}+a(Cit^{3-})\;↔\;M(Cit^{3-}{)}_{a}+nOH^{-}$$

In conclusion, the use of the C_6_H_5_Na_3_O_7_-containing NaNO_3_ mixed electrolyte during the TMEMM processing of the GH4169 alloy effectively reduces the generation of the insoluble electrolytic products. This improvement enhances the flow field mass transfer and current density distribution during TMEMM processing, thereby significantly improving the flatness of the non-machined zone on the surface of the GH4169 alloy.

**Figure 6:**
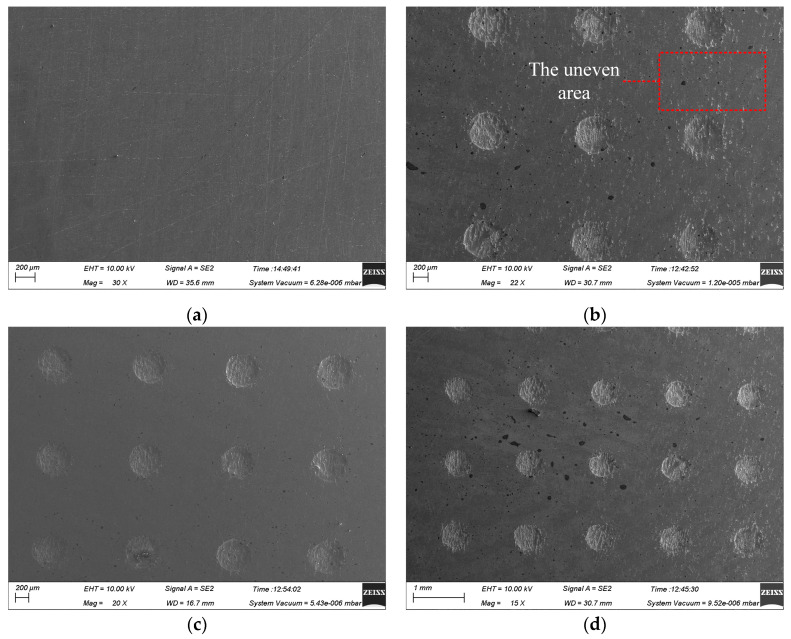
The morphology of machined micro-pit arrays of the GH4169 alloy under different concentrations of additives. (**a**) Initial workpiece surface; (**b**) 10 wt% NaNO_3_; (**c**) 10 wt% NaNO_3_ + 0.5 wt% C_6_H_5_Na_3_O_7_; and (**d**) 10 wt% NaNO_3_ + 1 wt% C_6_H_5_Na_3_O_7_.

Figure 6 shows the morphology of machined micro-pit arrays of the GH4169 alloy under different concentrations of C_6_H_5_Na_3_O_7_, with processing conditions of a 300 μm mask hole diameter and linear cathode dimension, a 12 V applied voltage, and a 0.1 r/min rotating speed of the cathode tool. As the concentration of the additive increases, the geometric profile of the micro-pit arrays and the surface quality of the non-machined zone first improve and then deteriorate. This effect is due to the formation of soluble metal-citrate complexes in the electrolyte, which reduce the formation and aggregation of insoluble electrolytic products, improving both the flow field mass transfer and the electric field distribution within the electrochemical machined zone. As a result, the surface flatness of the non-machined zone on the GH4169 alloy surface is significantly enhanced.

Thus, the addition of C_6_H_5_Na_3_O_7_ alleviates the non-uniform dissolution of the GH4169 alloy surface caused by the excessive accumulation of electrolytic products, leading to improved flatness in the non-machined zone during TMEMM processing. However, when the concentration of C_6_H_5_Na_3_O_7_ exceeds 1 wt%, the flatness decreases again. At high concentrations, excessive anodic polarization can accelerate the local dissolution of the workpiece surface [35]. Therefore, a 0.5 wt% C_6_H_5_Na_3_O_7_ + 10 wt% NaNO_3_ solution is chosen as the electrolyte for subsequent experiments.

**Figure 7:**
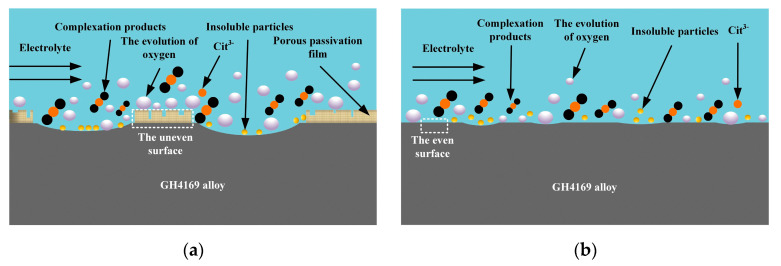
The influence mechanism of C_6_H_5_Na_3_O_7_ in the non-machined zone of the GH4169 alloy. (**a**) The initial reaction stage after the addition of C_6_H_5_Na_3_O_7_ and (**b**) the reaction completion stage after the addition of C_6_H_5_Na_3_O_7_.

Figure 7 illustrates the mechanism by which the C_6_H_5_Na_3_O_7_ influences the non-machined zone on the GH4169 alloy surface. Owing to the strong complexation ability of the Cit^3−^, the electrochemical dissolution process in the C_6_H_5_Na_3_O_7_-containing NaNO_3_ mixed electrolyte at low current density does not lead to the formation of insoluble electrolytic products on the surface of the non-machined zone (as shown in Figure 5b). As the electrochemical reaction progresses, the surface flatness of the GH4169 alloy tends to remain uniform.

#### 3.2.2. Influence of the Applied Voltage

**Figure 8:**
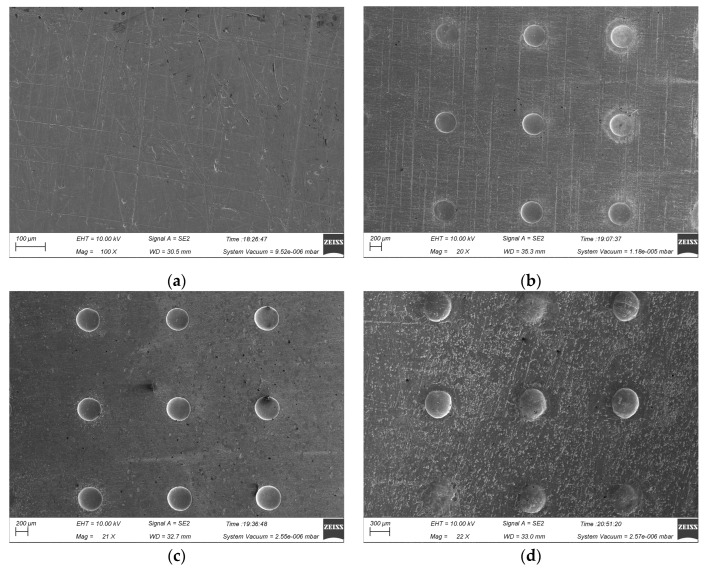
The impact of the applied voltages on the morphology of micro-pit arrays machined on the GH4169 alloy surface. (**a**) Initial workpiece surface; (**b**) U = 11V; (**c**) U = 12V; and (**d**) U = 13V.

Figure 8 shows the morphology of machined micro-pit arrays on the GH4169 alloy surface machined by roll-print mask electrochemical machining using the linear cathode at different applied voltages (11 V, 12 V, and 13 V). With a 11 V applied voltage, the etching depth of the micro-pit arrays is shallow, the inner surfaces are uneven, and significant stray corrosion is observed in the non-machined zone of the surface of the GH4169 alloy. As the applied voltage increases to 12 V, the micro-pit boundaries become more defined, displaying a regular circular contour, and the stray corrosion in the non-machined zone is minimal. When the applied voltage is further increased to 13 V, the current density in the inter-electrode gap is further increased, the intensity of the electrochemical reactions correspondingly intensifies. This heightened reaction rate leads to the substantial generation of electrolytic products, which subsequently accumulate at the base of the micro-pits. Such accumulation adversely affects the geometric integrity of the micro-pit arrays, causing a decline in their roundness. Concurrently, the bottom surfaces of these micro-pits exhibit increased roughness, which is indicative of a degradation in surface quality. This phenomenon is not confined to the machined areas; it also extends to the non-machined zones, where surface quality experiences a decline. Furthermore, the elevated current density exacerbates the occurrence of stray corrosion, making it more pronounced.

The uniformity of the size variation in micro-pit arrays in the process of roll-print mask electrochemical machining is commonly quantified through the coefficient of variation (CV). The CV is defined as the ratio of the size standard deviation to the average size. It is determined as the ratio of the standard deviation size to the mean size. A higher CV value indicates poorer uniformity in size distribution, as expressed by the following, see symbol description in Nomenclature:

(9) CV=δ/μ

**Figure 9:**
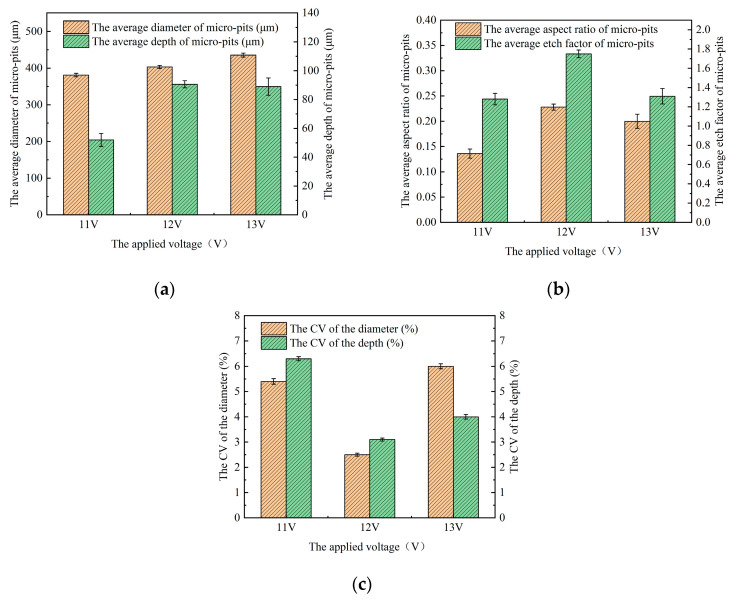
The size variation in micro-pit arrays at different voltages. (**a**) The variation in average depth and diameter of micro-pit arrays; (**b**) the variation in average aspect ratio and EF of micro-pit arrays; and (**c**) the CV of variation in the depth and diameter of the micro-pit arrays.

Figure 9 illustrates how the size variation and uniformity of the micro-pit arrays change with various applied voltages. As shown in Figure 9a, as the applied voltage increases from 11 V to 12 V, the mean depth and diameter of the micro-pit arrays gradually increase. This is due to the rising current density between the cathode and anode in the machined zone, leading to a higher electrochemical reaction rate over the same processing time, which results in a more substantial reaction and larger micro-pit diameters and depths. However, as the applied voltage further rises to 13 V, the radial corrosion of the micro-pits increases rapidly. As a result, while the average diameter of the micro-pit arrays increases significantly, the average depth increase is small, causing a significant reduction in the average aspect ratio and EF of the micro-pits (as shown in Figure 9b). The localization of the micro-pits also becomes worse. The reason is that the electrochemical reaction becomes more vigorous, rapidly increasing the quantity of the electrolytic products on the GH4169 alloy surface at excessively high voltages. They will accumulate at the bottom of the micro-pit arrays due to insufficient removal, while the radial corrosion continues, leading to a reduction in the aspect ratio and EF of the micro-pit arrays.

Figure 9c demonstrates that the CV values decrease as the applied voltage ranges from 11 V to 12 V, which contrasts with the trends observed for the depth and diameter of the micro-pit arrays. According to the TMEMM theory, at lower applied voltage, the current density is low, leading to an uneven electrochemical reaction rate within the machined zone. This results in a high CV and poor size uniformity in the micro-pit arrays. As the applied voltage increases, the current density in the electrochemical machined zone also increases, leading to a more uniform electrochemical reaction rate and to micro-pit arrays with improved size uniformity.

#### 3.2.3. Influence of the Rotating Speed of the Cathode Tool

In the process of roll-print mask electrochemical machining using the linear cathode, the rotating speed of the cathode tool influences the corrosion time, which subsequently has a significant effect on the development of micro-pit arrays. Therefore, studying the impact of the rotating speed of the cathode tool on the GH4169 alloy surface in roll-print mask electrochemical machining is crucial.

**Figure 10:**
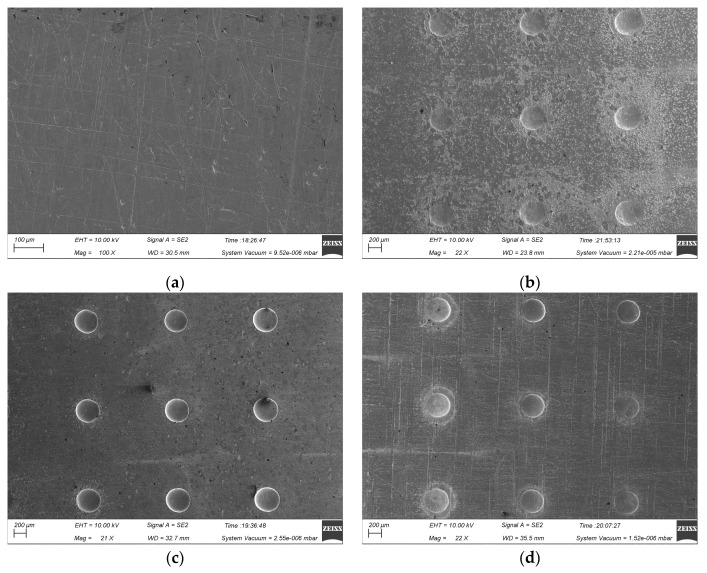
The impact of the rotating speeds of the cathode tool with respect to the configuration of micro-pit arrays. (**a**) Initial workpiece surface; (**b**) 0.05 r/min; (**c**) 0.1 r/min; and (**d**) 0.2 r/min.

Figure 10 presents the micro-pit array profiles on the GH4169 alloy surface processed with the linear cathode at various rotating speeds of the cathode tool (0.05 r/min, 0.1 r/min and 0.2 r/min). At a rotating speed of the cathode tool of 0.05 r/min, the micro-pits’ boundaries are irregular, and significant stray corrosion is observed around the edges. Increasing the rotating speed of the cathode tool to 0.1 r/min reduces the stray corrosion and results in more defined, regular, circular contours with smooth and flat micro-pit bottoms. However, at a rotating speed of the cathode tool of 0.2 r/min, the electrochemical reaction time will be significantly shortened, and the surface of the anode workpiece will not be fully dissolved, so that the average etching depth of the micro-pit arrays decreases, and boundary deformation occurs, leading to poorer quality micro-pit arrays.

A higher rotating speed of the cathode tool reduce the corrosion time, which weakens the electrochemical dissolution reaction within the machined zone, resulting in less rounded micro-pits boundaries and shallower micro-pit arrays. Conversely, as the rotating speed of the cathode tool decreases, the electrochemical reaction time increases, improving the boundary circularity of the micro-pits. However, if the rotating speed of the cathode tool is reduced excessively, leading to prolonged corrosion times, the electrolytic products accumulate at the bottom of the micro-pit arrays due to the inadequate flow field mass transfer, while radial corrosion continues. This accumulation exacerbates the decline in the localization accuracy and overall micro-pit array manufacturing quality.

**Figure 11:**
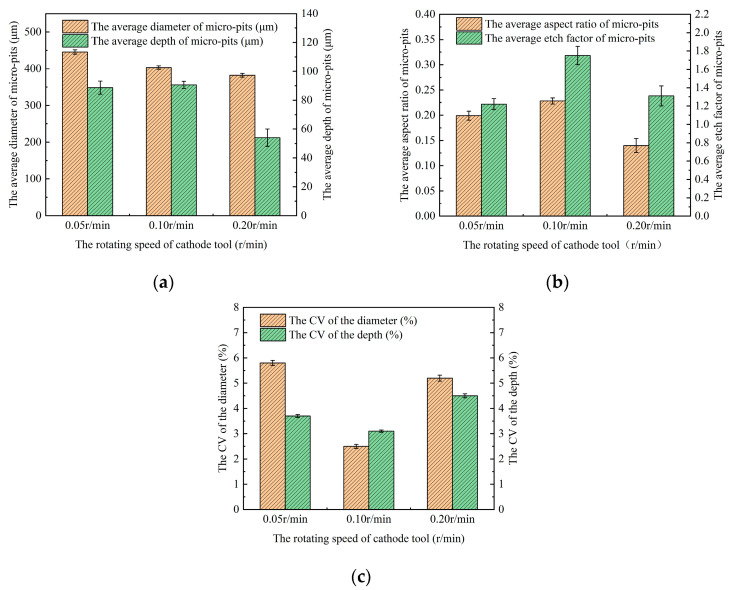
The size variation in micro-pit arrays under different rotating speeds of the cathode tool. (**a**) The variation in the average diameter and depth of micro-pit arrays; (**b**) the variation in the average aspect ratio and EF of micro-pit arrays; and (**c**) the CV of variation in the diameter and depth of the micro-pit arrays.

Figure 11 illustrates the effects of different rotating speeds of the cathode tool on the size variation and uniformity of the micro-pit arrays. As the rotating speeds of the cathode tool increases, the actual corrosion time of the electrochemical reaction zone decreases, while the radial etching time of the micro-pits continues. The mean diameter of the micro-pits decreases from 445.5 μm to 382 μm, whereas the average depth of the micro-pits initially increases from 88.68 μm to 90.6 μm and then decreases sharply to 54 μm.

This can be explained by the fact that the rate of the electrolytic product formation exceeds the electrolyte erosion rate at lower rotating speeds of the cathode tool, leading to the accumulation of electrolytic products at the bottom of the micro-pit arrays, which cannot be discharged in time. As a result, the axial etching of the micro-pits stagnates while the radial corrosion continues. Consequently, the average aspect ratio and EF are low, and the localization and uniformity of the micro-pit arrays are poor. When the rotating speed of the cathode tool increases to 0.1 r/min, the formation rate of the electrolytic products is basically equal to the electrolyte erosion rate, allowing the electrochemical reaction to proceed fully, leading to improved quality micro-pit arrays, a larger average aspect ratio and EF, and better localization and uniformity. However, at the excessively high rotating speed of the cathode tool of 0.2 r/min, the actual corrosion time becomes insufficient for the electrochemical reaction to fully develop, resulting in shallow micro-pits with an average depth of only 54 μm. Correspondingly, the average aspect ratio and EF decrease, which illustrates that the localization and uniformity of the micro-pit arrays show some decrease. These findings align with the results shown in Figure 10.

## 4. Conclusions

This study investigates roll-print mask electrochemical machining using linear cathode micro-pit arrays on the GH4169 alloy surface. The effects of the composition of the C_6_H_5_Na_3_O_7_-containing NaNO_3_ mixed electrolyte, the concentration of the C_6_H_5_Na_3_O_7_, the applied voltage, and the rotating speeds of the cathode tool on the morphology and profile of micro-pit arrays on the GH4169 alloy surface were studied experimentally. Reflecting on the experimental findings, the following conclusions are deduced:

1. Compared with the single NaNO_3_ solution, the mixed electrolyte containing C_6_H_5_Na_3_O_7_ is more suitable for TMEMM of the micro-pit arrays on the GH4169 alloy surface, significantly improving the localization and geometric morphology of the micro-pit arrays;
2. The additive concentration of 0.5 wt% C_6_H_5_Na_3_O_7_ effectively reduces the stray corrosion in the non-machined zone. However, both excessively high and low concentrations of C_6_H_5_Na_3_O_7_ are detrimental to minimizing stray corrosion. Additionally, the appropriate applied voltage and rotating speeds of the cathode tool enhance the uniformity and localization of the micro-pit arrays;
3. The optimal electrochemical machining parameters are 0.5 wt% C_6_H_5_Na_3_O_7_ + 10 wt% NaNO_3_ mixed electrolyte, a 12 V applied voltage, and a 0.1 r/min rotating speed of the cathode tool; we ascertained this through the comparative analysis of the geometric morphology and geometric size distribution of the micro-pit arrays under the corresponding process parameters. Under these conditions, the machined micro-pit arrays achieved a diameter of 405.85 ± 9.45 μm, a depth of 87.5 ± 8.5 μm, an average aspect ratio of 0.22, an average EF of 1.67, the CV of the diameter of 0.01, and the CV of the depth of 0.06.

To sum up, roll-print mask electrochemical machining using the linear cathode can effectively improve the localization and uniformity of the micro-pit arrays on the GH4169 alloy surface, which provides a promising processing method for improving the performance and service life of gas turbines for space shuttles and rocket engines.

## Figures and Tables

**Table 1:** Chemical composition of 304 SS and GH4169 alloy.

Workpiece Material	Composition (%)									
	Ni	Cr	Mn	Si	C	Fe	Nb	Mo	Ti	Al
304 SS	8.92	18.38	2.00	1.00	0.08	Balance	-	-	-	-
GH4169 alloy	52.75	17.96	0.12	0.16	0.038	Balance	5.13	3.05	1.09	0.55

**Table 2:** Main fabrication conditions and process parameters.

Process Parameter	Value
Electrolyte	H_2_SO_4_, NaOH, NaNO_3_, NaCl, C_6_H_5_Na_3_O_7_-containing NaNO_3_ electrolyte
Electrolyte concentration	10 wt%
C_6_H_5_Na_3_O_7_ addition agent	0.5 wt%, 1 wt%
The anode material	GH4169 alloy
The line cathode	0.3 mm Cu wire (diameter)
The mask hole diameter	0.3 mm
The rotating speed of cathode tool	0.05, 0.1, 0.2 r/min
Inter-electrode gap	0.1 mm
Applied voltage	11 V, 12 V, 13 V
The flow rate of electrolyte	1 L/min

## Data Availability

The original contributions presented in the study are included in the article, further inquiries can be directed to the corresponding author/s.

## Nomenclature

δ	The standard deviation of size
μ	The mean of size
